# Characteristics of Human and Microbiome RNA Profiles in Saliva

**DOI:** 10.1080/15476286.2023.2229596

**Published:** 2023-07-03

**Authors:** Fangjia Tong, Gongyu Tang, Xiaowei Wang

**Affiliations:** a Department of Pharmacology and Regenerative Medicine, University of Illinois at Chicago, Chicago, IL, USA; b University of Illinois Cancer Center, Chicago, IL, USA; c Department of Mechanical Engineering and Materials Science, Washington University in St. Louis, St. Louis, MO, USA

**Keywords:** saliva, Small RNA-seq, microbiome, exosome, microvesicle

## Abstract

Saliva is a convenient non-invasive source of liquid biopsy to monitor human health and diagnose diseases. In particular, extracellular vesicles (EVs) in saliva can potentially reveal clinically relevant information for systemic health. Recent studies have shown that RNA in saliva EVs could be exploited as biomarkers for disease diagnosis. However, there is no standardized protocol for profiling RNA in saliva EV nor clear guideline on selecting saliva fractions for biomarker analysis. To address these issues, we established a robust protocol for small RNA profiling from fractionated saliva. With this method, we performed comprehensive small RNA sequencing of four saliva fractions, including cell-free saliva (CFS), EV-depleted saliva (EV-D), exosome (EXO), and microvesicle (MV) from ten healthy volunteers. By comparing the expression profiles of total RNA from these fractions, we found that MV was most enriched in microbiome RNA (76.2% of total reads on average), whereas EV-D was notably enriched in human RNA (70.3% of total reads on average). As for human RNA composition, CFS and EV-D were both enriched in snoRNA and tRNA compared with the two EV fractions (EXO and MV, *P* < 0.05). Interestingly, EXO and MV had highly correlated expression profiles for various noncoding RNAs such as miRNA, tRNA, and yRNA. Our study revealed unique characteristics of circulating RNAs in various saliva fractions, which provides a guideline on preparing saliva samples to study specific RNA biomarkers of interest.

## Introduction

In recent years, emerging methods based on liquid biopsy have shown great potential for disease diagnosis, therapeutic guidance, and recurrence monitoring [1,2]. Among all analytes of liquid biopsy, extracellular vesicles (EVs) have drawn enormous attention as effective carriers for intercellular communications. EVs are lipid-bound vesicles secreted into the extracellular space by cells [3]. Two primary forms of EV are exosome (EXO) and microvesicle (MV). The size of EXO is 30–150 nm which is smaller than MV. They have distinct characteristics based on their biogenesis, release mechanisms, and biological activities [4]. Many studies have reported that EVs could be exploited as biomarkers for disease diagnosis [5].

Among EV-focused liquid biopsies, saliva offers unique advantages due to its noninvasiveness, ease of collection, cost-effectiveness, and safety [6]. Moreover, saliva reflects both systemic and local health and allows for real-time monitoring of dynamic changes. Saliva contains a diverse range of molecules that play critical roles during disease development, making it an ideal resource for identifying biomarkers for the diagnosis, monitoring, and prognosis of various human diseases. For example, studies have shown that the level of miR-423-5p in saliva is a promising diagnostic and prognostic biomarker for oral cancer [7]. Additionally, the levels of miR-17 and miR-21 in saliva have shown potential applications in pancreatic cancer detection [8]. Saliva has been intensely studied for developing biomarkers to monitor a variety of human diseases [9,10] however, few studies have focused specifically on characterization of EVs in saliva [10]. Discovering the composition and functional roles of EV in saliva is important to reveal the underlying regulatory mechanisms for disease development and progression.

Despite the advantages of saliva EV as potential disease biomarkers, advancement in this field is hampered by major technical obstacles such as the lack of standardized protocol for RNA profiling, and guidance on saliva sample processing for biomarker analysis. To address these issues, we established an improved protocol for efficient isolation of saliva RNA and further performed small RNA-seq profiling to characterize multiple saliva fractions including cell-free saliva (**CFS**), EV-depleted saliva (**EV-D**), EXO, and MV from ten healthy volunteers. In this way, we identified common and unique characteristics of the RNA profiles across different saliva fractions, which provides a better understanding of specific RNA profiles associated with each saliva fraction. Thus, our work would offer valuable guidelines for researchers in preparing and selecting the most suitable saliva fraction for their biomarker studies, based on specific RNA of interest. This knowledge would contribute to enhancing the accuracy and effectiveness of saliva-based biomarker investigations.

## Results

### Establishing a robust method for RNA extraction from saliva

**Figure 1.**
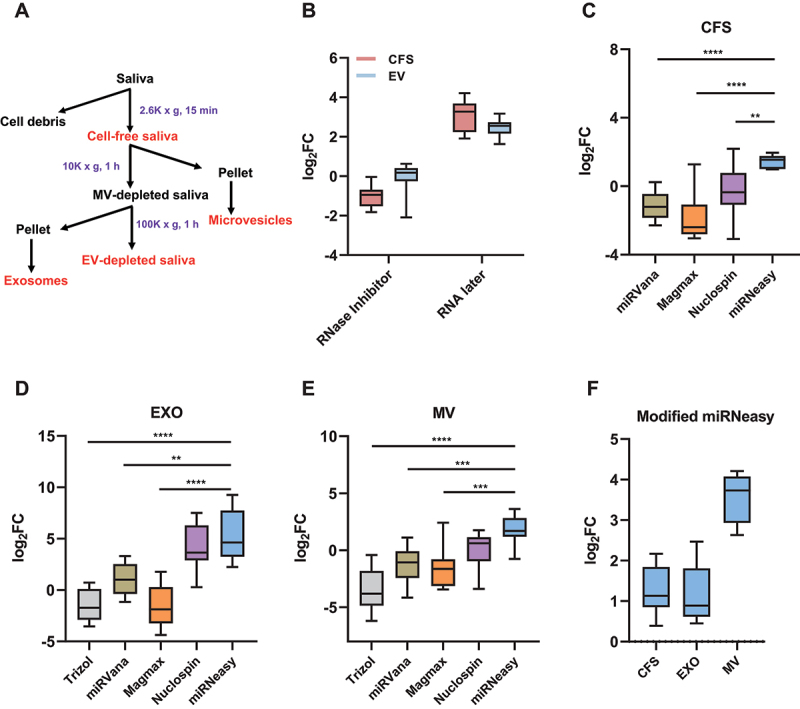
Establishing an improved method for RNA extraction from individual saliva fractions. (A) Flowchart showing the times and speeds of centrifugation for the isolation of four saliva fractions. (B) Comparing the effectiveness of two RNA preservative methods. (C-E) Comparison of five RNA extraction methods for CFS (C), EXO (D), and MV (E). (F) Comparison between standard miRNeasy and modified miRNeasy protocols. The results represent Ct value changes summarized for ten representative miRNAs. miRNA quantitation was performed by RT-qPCR. CFS, cell-free saliva; EXO, exosome; MV, microvesicle.

We compared two common saliva preprocessing methods involving RNAlater and RNase inhibitor, respectively. Specifically, we evaluated isolated RNA amount from these samples with aforementioned RNA preservatives. To this end, relative quantity of ten miRNAs (see Methods for details) were determined by RT-qPCR. As shown in Figure 1B, when compared to no treatment control, the addition of RNAlater during saliva collection resulted in higher RNA yield in the CFS and EV fractions, with an average log_2_FC of 3.1 and 2.5, respectively. In contrast, the addition of RNase inhibitor did not improve the RNA yield as compared to no treatment control. Based on this result, RNAlater was included in our standard protocol for saliva preprocessing.

*P < 0.05, **P < 0.01, ***P < 0.001, ****P < 0.0001.

Next, we compared five widely used methods for RNA extraction from individual saliva fractions. For the CFS fraction, we used Trizol LS extraction protocol as reference standard for comparing the yield of ten miRNAs as mentioned above. RT-qPCR indicated that miRNeasy resulted in the highest RNA yield with an average log_2_FC of 1.4 (Figure 1C). As for the EV fractions (EXO and MV), these same methods were compared with no extraction control (Figure 1D-E). Similar to CFS analysis, miRNeasy showed the best performance in isolating RNA from EXO and MV, with an average log_2_FC of 5.2 and 1.7, respectively. Besides miRNeasy, Nucleospin had the second-best performance, while Trizol LS and MagMAX showed lower yields compared with no extraction control. In conclusion, miRNeasy outperformed the other methods and provided a higher RNA yield for saliva fractions. Thus, miRNeasy was adopted as the standard protocol for saliva RNA extraction in our study. Based on the miRNeasy protocol, we tested multiple modifications for further improving the RNA yield. To this end, we evaluated the effects of modifications, which involved glycogen co-precipitant and a second round of chloroform extraction. Comparative RT-qPCR analysis indicated these modifications resulted in improved RNA recovery for all included saliva fractions. Impressively, the RNA yield for MV was improved by log_2_FC of 3.6 (Figure 1F).

## Microbiome diversity across different saliva fractions

**Figure 2.**
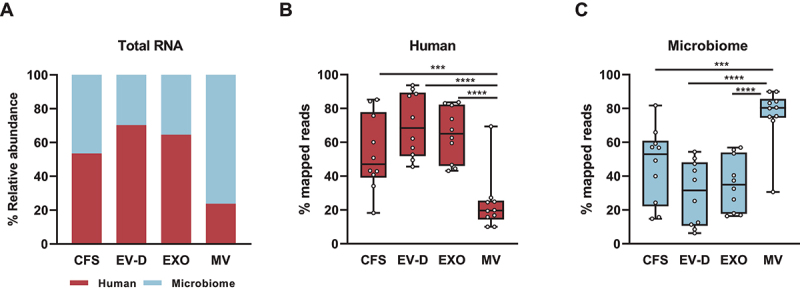
Total RNA composition of CFS, EXO, MV, and EV-D. (A) Relative composition of total RNA across individual saliva fractions. (B) Box plot for the percentage distribution of human RNA across saliva fractions at individual donor level. (C) Box plot for the percentage of microbiomes across saliva fractions at individual donor level. CFS, cell-free saliva; EXO, exosome; MV, microvesicle; EV-D, EV-depleted saliva.

We performed small RNA sequencing to profile the RNA in four saliva fractions (CFS, EV-D, EXO, and MV) from ten healthy volunteers. First, we determined the relative distribution of human and microbiome RNA from these individuals (Figure 2A). Human RNA was abundantly present in CFS, EV-D, and EXO, representing 53.5%, 70.3%, and 64.6% of total mapped reads, respectively. In contrast, MV had the highest percentage of microbiome reads, representing 76.2% of total reads on average. We further examined the differences in RNA distribution across specific saliva fractions at the individual level. As shown in Figure 2B, EV-D contained a significantly higher percentage of human reads, which was consistent across all ten individuals. As for microbiome reads, MV had the highest abundance across all saliva fractions in most individuals (nine out of ten as shown in Figure 2C).

***P < 0.001, ****P < 0.0001.

**Figure 3.**
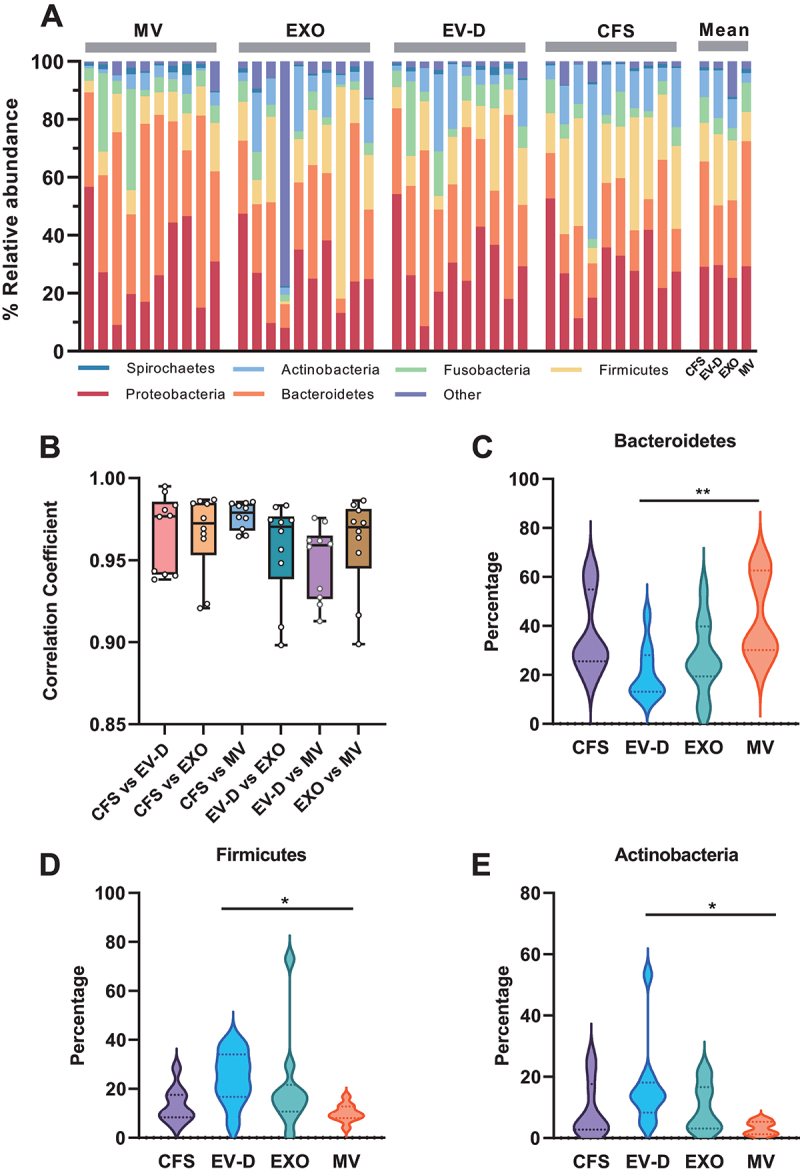
The microbiome profiles of CFS, EXO, MV, and EV-D. (A) The microbiome composition of individual saliva fractions at the phylum level. (B) Distributions of pairwise correlation coefficients across four saliva fractions. (C-E) Violin plot depicting relative expression levels of *Bacteroidetes* (C), *Firmicutes* (D), and *Actinobacteria* (E) in individual saliva fractions. CFS, cell-free saliva; EXO, exosome; MV, microvesicle; EV-D, EV-depleted saliva.

Given the abundance of microbiome reads in our sequencing data, we further compared the taxonomic characteristics of microbiome in specific saliva fractions. As shown in Figure 3A, we summarized the microbiome composition of all individuals at the phylum level. Major phyla identified include *Spirochaetes*, *Actinobacteria*, *Fusobacteria*, *Firmicutes*, *Proteobacteria*, and *Bacteroidetes*. Interestingly, the microbiome composition was considerably variable among individuals as well as across saliva fractions. Specifically, CFS and MV had higher percentages of *Bacteroidetes* compared with EV-D and EXO, representing 36.3% and 43.2% of total microbiome reads, respectively. In contrast, EV-D and EXO had higher percentages of *Firmicutes* (24.6% and 20.6%, respectively), whereas *Proteobacteria* was similarly expressed across all four saliva fractions (28.4% on average).

*P < 0.05, **P < 0.01.

We further performed pairwise correlative analysis of the microbiome profiles across different saliva fractions (Figure 3B). The highest correlation was found between CFS and MV (Pearson *r* = 0.96–0.98 among the ten individuals), whereas the lowest correlation was found between EV-D and MV (*r* = 0.91–0.98). Next, we analysed specific microbiome phyla that were differentially expressed across these fractions. *Bacteroidetes* was identified as the most prevalent phylum in MV as compared to EV-D (Figure 3C). In contrast, *Firmicutes* and *Actinobacteria* showed the opposite pattern, with more reads detected in EV-D than MV (Figure 3D-E). In summary, our work indicated that microbiome reads were abundantly present in all four saliva fractions, however, with significant taxonomic diversity across individuals and saliva fractions.

## Individual saliva fractions had distinct human RNA profiles

**Figure 4.**
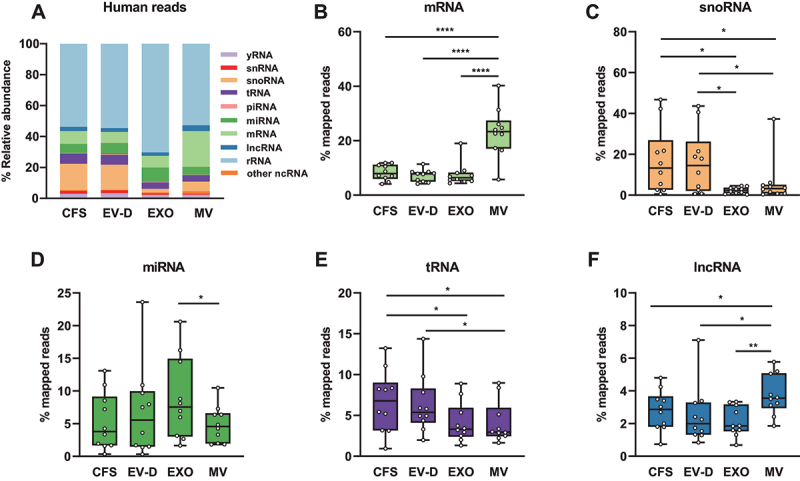
Human RNA profiles of CFS, EXO, MV, and EV-D. (A) Composition of human RNAs in individual saliva fractions. (B-F) Percentage distributions of mapped reads in four saliva fractions for mRNA (B), snoRNA (C), miRNA (D), tRNA (E), and lncRNA (F). CFS, cell-free saliva; EXO, exosome; MV, microvesicle; EV-D, EV-depleted saliva.

To determine the expression profile of human RNAs, we mapped the human reads to available database annotations of human RNAs, including mRNA, miRNA, tRNA, yRNA, piRNA, ribosomal RNA (rRNA), long non-coding RNA (lncRNA), small nucleolar RNA (snoRNA), small nuclear RNA (snRNA), and other non-coding RNA (ncRNA) (Figure 4A). CFS had a comparable RNA profile to EV-D while showing distinct patterns from the EV fractions. Among all RNA species, rRNA was the most abundant, accounting for 53.7%, 54.5%, 70.3%, and 52.7% in CFS, EV-D, EXO, and MV, respectively.

*P < 0.05, **P < 0.01, ***P < 0.001, ****P < 0.0001.

Next, we performed differential expression analysis by comparing the RNA profiles of all fractions across individual subjects (Figure 4B-F). Compared with MV, all other fractions contained significantly lower levels of mRNA (7.7% vs. 23.1% on average) (Figure 4B). On the other hand, CFS and EV-D showed significantly higher levels of snoRNAs when compared with the EV fractions (17.0%, 16.2% vs. 4.3% on average) (Figure 4C). EXO had the highest level of miRNAs among the four fractions, representing 9.4% of total human reads on average (Figure 4D). Other noticeable differences were tRNA enrichment in both CFS and EV-D, and lncRNA enrichment in MV (Figure 4E-F). In summary, CFS and EV-D showed similar human RNA expression profiles, both of which were distinct from the EV fractions. Thus, different saliva fractions demonstrate correlated but distinct expression profiles for various human RNA species.

## MV and EXO shared similar miRNA expression profiles

**Figure 5.**
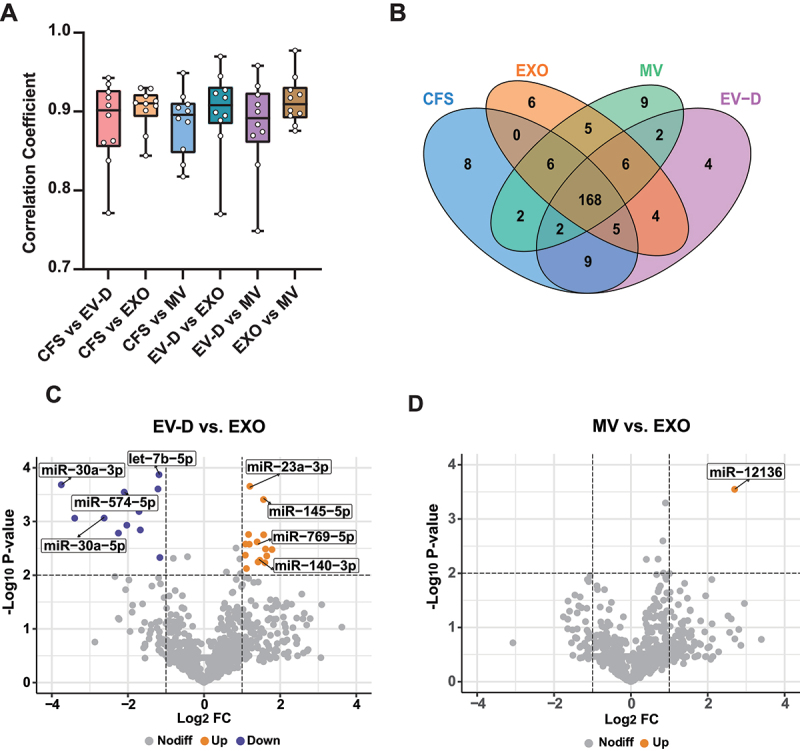
miRNA expression profiles of CFS, EXO, MV, and EV-D. (A) Distributions of pairwise correlation coefficients in ten individual donors. (B) Venn diagram to summarize unique and common miRNAs across all saliva fractions. Two hundred most abundant miRNAs are presented. (C-D) Volcano plots for differentially expressed miRNAs between EV-D and EXO (C), and between MV and EXO (D). CFS, cell-free saliva; EXO, exosome; MV, microvesicle; EV-D, EV-depleted saliva.

As previously described, miRNAs were significantly enriched in EXO. We further investigated the miRNA profiles across the four saliva fractions. First, we performed pairwise correlative analysis of miRNA expression profiles, with correlation coefficients between fractions or individuals ranging from 0.75 to 0.98 (Figure 5A). Among all fractions, EXO and MV demonstrated the strongest correlation (r = 0.91 on average). The correlation between CFS and EXO were comparable to those between EV-D and EXO (r = 0.90 on average). Similarly, CFS, EV-D, and MV had correlated profiles, with *r* values of 0.88 on average.

Next, we evaluated unique and overlapping miRNAs in the four fractions among 200 most abundantly expressed miRNAs (Figure 5B). We observed 168 common miRNAs and 27 unique miRNAs across the four fractions. Previous studies reported that specific miRNAs were sorted into EXO via four potential mechanisms, such as the nSMase2-dependent pathway [11]. To further investigate EXO-specific miRNAs, we compared differentially expressed miRNAs in EXO as compared with the other three fractions. As shown in Figure 5C, for pairwise analysis, a total of 28 miRNAs were found differentially expressed between EV-D and EXO, with fifteen and thirteen miRNAs enriched in EV-D and EXO, respectively (Supplementary Table S1). Likewise, five and thirteen miRNAs were found enriched in CFS and EXO, respectively (Supplementary Table S2). In contrast, when MV and EXO were compared, only one differentially expressed miRNA (miR-12136) was found enriched in MV (Figure 5D). Interestingly, we found seven miRNAs specifically enriched in both EXO and MV, and two miRNAs specifically enriched in both CFS and EV-D (Supplementary Figure S1). In summary, EXO and MV shared similar miRNA profiles, both of which were distinctively different from CFS and EV-D.

## Distinct profiles for tRNA fragments (tRfs) among different saliva fractions

**Figure 6.**
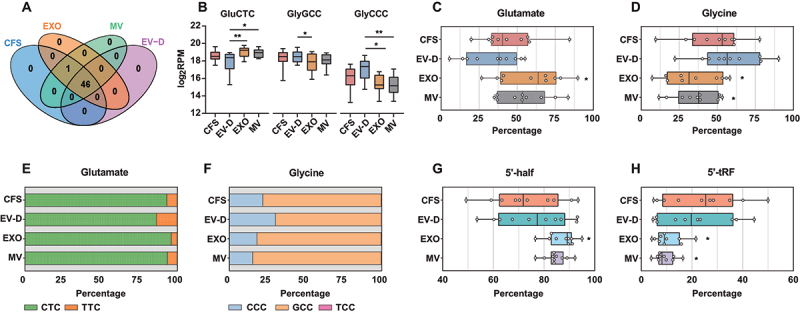
tRF expression profiles of CFS, EXO, MV, and EV-D. (A) Venn diagram to summarize unique and common tRFs across saliva fractions. (B) Relative expression levels of three most abundant tRFs in individual saliva fractions. (C-D) Box plot depicting the percentage distribution of tRFs across saliva fractions at individual donor level for glutamate (C), and glycine (D). (E-F) Composition of tRFs in individual saliva fractions for glutamate (E), and glycine (F). (G-H). Box plot depicting the percentage distribution of tRFs across saliva fractions at individual donor level for 5’-half tRFs (G) and 5’-tRFs (H). CFS, cell-free saliva; EXO, exosome; MV, microvesicle; EV-D, EV-depleted saliva; tRF, tRNA fragment.

Compared with the EV fractions, CFS and EV-D showed significantly higher levels of tRNA expression (Figure 4E). Interestingly, all obtained tRNA reads were derived from tRFs instead of full-length tRNAs. Thus, we further analysed the tRF profiles by evaluating unique and overlapping tRFs. The four saliva fractions shared almost the same tRFs (46 out of 47) with only one not present in EV-D (Figure 6A). In particular, we found that GluCTC, GlyGCC, and GlyCCC were the most abundant tRFs across all fractions. As shown in Figure 6B, GluCTC was significantly enriched in EXO compared with EV-D, whereas GlyGCC and GlyCCC were enriched in EV-D.

*P < 0.05, **P < 0.01.

We further categorized tRFs based on their matched amino acid. Glutamate tRFs were predominant in EXO and MV, representing 58.4% and 54.4% on average, respectively (Figure 6C); glycine tRFs were predominant in CFS and EV-D, representing 48.3% and 58.2% on average, respectively (Figure 6D). As multiple anticodons can encode the same amino acid, we investigated the relative abundance of all known anticodons (Figure 6E-F). The percentage distribution of each anticodon varied across saliva fractions. Of note, EV-D was enriched in tRFs containing TTC anticodon for glutamate and CCC for glycine. On the other hand, CFS and MV had tRF profiles with comparable anticodon usage for glutamate, whereas EXO and MV shared similar anticodon profiles for glycine.

Next, we categorized tRFs based on fragmentation patterns. We found that 5’-half tRF was the most abundant type across all four fractions, followed by 5’-tRF (Figure 6G-F). EXO and MV had higher percentages of 5’-half tRF compared with CFS and EV-D (85.8% vs. 74.5% on average, respectively; Figure 6G). On the other hand, 5’-tRF was more enriched in CFS and EV-D (Figure 6H). These results suggest that EXO and MV had comparable tRF expression profiles, which differed from CFS and EV-D.

## Distinct profiles for yRNA fragments among different saliva fractions

**Figure 7.**
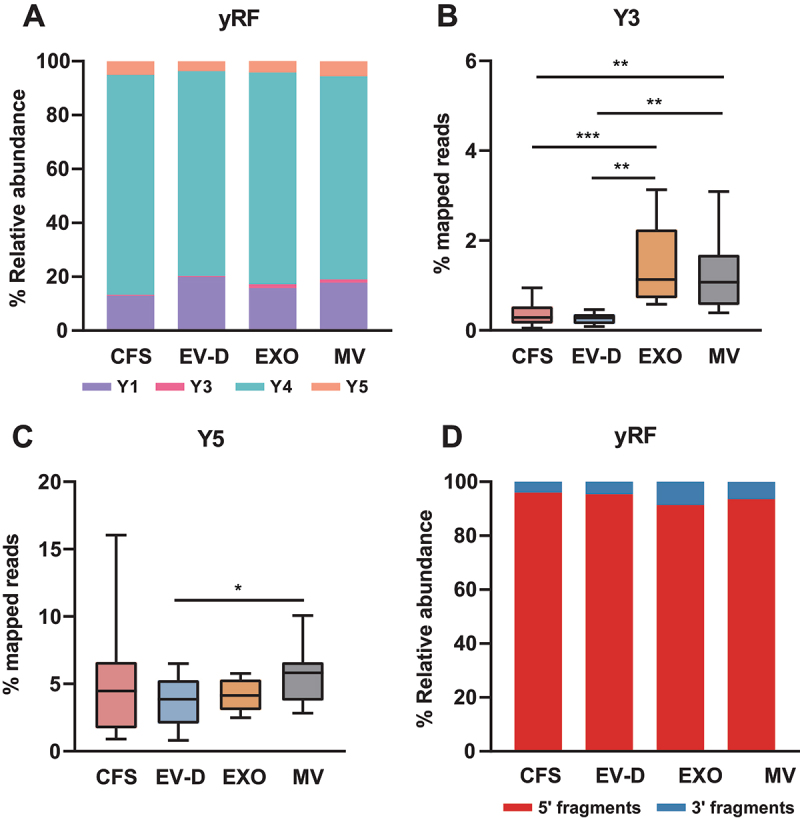
yRF expression profiles of CFS, EXO, MV, and EV-D. (A) Composition of yRFs in individual saliva fractions. (B-C) Percentage distribution of mapped reads in four saliva fractions for Y3 (B) and Y5 (C). (D) Composition of yRFs in individual saliva fractions by fragmentation pattern. CFS, cell-free saliva; EXO, exosome; MV, microvesicle; EV-D, EV-depleted saliva; yRF, yRNA fragment.

As shown in Figure 4A, we identified 3% of human reads that were mapped to yRNA. All mapped yRNA reads were derived from yRNA fragments (yRFs). Recent studies showed that yRFs play an important role in inter-cellular signalling as well as disease development [12]. In our analysis, we further compared yRF expression profiles across all saliva fractions. As shown in Figure 7A, most reads were mapped to Y4 in all fractions (77.9% on average), followed by Y1 and Y5 (16.6% and 4.7% on average, respectively). EXO and MV showed significantly higher percentages of Y3 compared to CFS and EV-D (Figure 7B). MV had the highest percentage of Y5, whereas EV-D had a significantly lower level of Y5 compared with MV (Figure 7C). We also categorized yRNA reads based on fragmentation patterns. As shown in Figure 7D, CFS and EV-D showed similar fragment composition, with predominantly 5’-fragments across all fractions.

*P <0.05, **P <0.01, ***P <0.001.

## Discussion

In this study, we have developed an improved small RNA-seq protocol for profiling different saliva fractions. Compared with other fractions, MV was highly enriched in microbiome RNA, which could be explained by the pelleting of bacterial cells during MV isolation [13]. Interestingly, taxonomic characteristics of the microbiomes differ significantly across saliva fractions. In particular, *Bacteroidetes* were the most abundant phylum in CFS and MV, whereas *Firmicutes* were most abundant in EV-D and EXO. *Bacteroidetes* and *Firmicutes* were the main phyla presented in human saliva [14]. It has been reported that *Bacteroidetes* were negatively correlated with disease activity in ulcerative colitis, and *Firmicutes* levels in saliva reflected the gut condition [15,16]. In addition, the *Firmicutes*/*Bacteroidetes* ratio could serve as an indicator of obesity development [17]. Thus, the relative abundance of a phylum could potentially be used as an indicator of human health. Our work indicated potential application of microbiome RNA profiles in saliva, as well as their taxonomic diversity across individual saliva fractions.

As for human RNA composition, we found that rRNA was the most abundant RNA species across all saliva fractions, especially in EXO. Many studies reported the enrichment of rRNAs in EXO, which is consistent with our findings [18,19]. In our study, all mapped rRNA reads were derived from rRNA fragments. Multiple studies reported that rRNA fragments may function similarly to miRNAs by suppressing mRNA expression or protein translation [20–22]. Further studies have shown diverse roles of rRNA fragments in regulating metabolism, cell viability and proliferation [23,24]. Hence, highly enriched rRNA fragments in the circulation could mediate intercellular communications that are relevant to human health and diseases.

The miRNA expression profiles in EXO have been intensely studied in recent years and many studies indicate that exosomal miRNAs could be used as biomarkers for human diseases. Compared with other saliva fractions, miRNAs were most abundant in EXO. The miRNA expression profiles of EXO and MV are highly correlated. Although EXO and MV differ in many aspects, they both feature EV-specific characteristics, such as membrane-bound vesicles, cargo selection for packaging, and cargo transport across cells [25]. For these reasons, EXO and MV showed similarities in miRNA expression, particularly when compared with CFS or EV-D. In addition, we also found a set of EV-specific miRNAs in saliva, such as miR-30a-5p, miR-125a-5p, and miR-200a-3p, implicating functional roles of these miRNAs in intercellular communications. As supporting evidence, exosomal miR-30a could regulate autophagy in a paracrine-like manner in ischaemic heart disease [26] and promote tumour metastasis in colon cancer [27].

In contrast to miRNAs, snoRNAs were significantly enriched in CFS and EV-D as compared with the EV fractions. snoRNAs are typically found in the nucleoli and are mainly responsible for rRNA posttranscriptional modification and maturation [28]. Interestingly, multiple studies reported snoRNA depletion in EVs isolated from plasma [29,30]. In our previous study, snoRNAs were similarly depleted in EVs derived from cancer cells [31]. Therefore, the depletion of snoRNAs in the EV fractions might be a general phenomenon resulting from EV-specific RNA selection and packaging processes.

Similar to snoRNAs, tRNA fragments (tRFs) were also found enriched in CFS and EV-D. The main role of full-length tRNAs is to act as key adaptor molecules for peptide elongation during translation. We found these tRNA reads were all derived from tRFs in our small RNA-seq libraries. tRFs are generated by specific cleavage of primary and mature tRNAs. Among all tRFs, two major subgroups including 5’- half tRFs and 5’-tRFs were most abundant in saliva fractions. Increasing evidence indicated that tRFs play important functions in human disease development [32,33]. Thus, changes in saliva tRF profiles could serve as potential disease biomarkers. For example, GluCTC, the most abundant tRF species in our study, was reported as a diagnostic biomarker for liver cancer [34]. In addition, GluCTC was associated with chemotherapy outcomes in various cancers [35].

Previous studies revealed that yRNAs and yRFs play important roles in disease development [12]. yRNAs are highly conserved non-coding RNAs, and four yRNAs have been identified to date, including Y1, Y3, Y4, and Y5. We found Y4 to be the most abundant yRNA in saliva. Our data indicated the yRNA reads were all derived from yRFs. yRFs are produced in apoptotic cells resulting from yRNA degradation through a caspase-3 dependent process [12]. yRFs can be broadly classified into two groups: 5’-fragments and 3’-fragments. We found that 5’-fragments were the most abundant, representing over 90% of yRFs. The functions of yRFs have not been well defined. Several studies hypothesized that yRFs may undergo a similar biogenesis process shared with miRNAs because of their characteristic stem-loop structures [36]. Same with miRNAs, yRFs are transported from nucleus to cytoplasm through Exportin 5 and Ran GTPase. However, yRFs do not bind to Ago2 RISC complex for regulating target expression [37,38]. They can regulate apoptosis and promote inflammation in macrophages by activating caspase 3 and NF-κB signalling pathways [39]. In addition, exosomal yRFs were found to suppress cardiomyocyte death by inducing IL-10 release and reducing TNFα levels [40]. Given the diverse roles of yRFs, it would be interesting to further investigate their implications in disease development.

While we provided a comprehensive analysis of distinct small RNA profiles in four saliva fractions, there are some limitations in this study. Firstly, we did not compare differential ultracentrifugation with other commercial EV isolation methods. To date, differential ultracentrifugation is widely considered as the gold standard for EV isolation, and it would be important to compare its performance with alternative methods. Although our primary focus was on RNA isolation, future studies should consider evaluating and comparing various EV isolation methods. Secondly, we only focused on relative changes in small RNA profiles across different saliva fractions, without performing absolute RNA quantitation. To this end, incorporating spike-in controls in future studies would enable more precise comparisons for absolute quantitation across different saliva fractions. Additionally, we did not analyse the mRNA species in this study. Our focus was on small RNA species by small RNA-seq analysis. Thus, data on mRNA from our sequencing results were limited; total RNA-seq in future studies would be helpful to delineate the expression profiles of mRNA or other long RNA species, thereby providing a complete picture of all RNA species in saliva.

In summary, we have established an improved small RNA profiling protocol for saliva study. Based on our findings, saliva, especially the MV fraction, may serve as a useful resource for oral microbiome study; EXO or EV-D, are more suited for profiling human RNAs; CFS can be an optimal resource for balanced study of both human RNAs and microbiomes. This study presents unique characteristics of various circulating RNAs in saliva, which provides a guideline on selecting specific saliva fractions for targeted biomarker discovery.

## Materials and methods

### Ethics statement

All the participants provided written informed consent to participate in the study. This study was approved by the Institutional Review Board of the University of Illinois Chicago.

## Saliva collection and preprocessing

Saliva was collected from ten healthy volunteers who were asked to refrain from drinking, eating, or chewing anything for at least 30 min before sample collection. For each volunteer, about 5 mL of unstimulated saliva was collected, and then mixed with 1 mL of RNAlater (Thermo Fisher). Two sample processing methods were assessed for initial treatment of saliva. Specifically, an aliquot of saliva was treated with either RNAlater or RNase Inhibitor (Thermo Fisher), and then centrifuged at 2600 × *g* for 15 min at 4°C to remove cells and debris. Cell-free saliva supernatant was then used for EXO and MV isolation or direct extraction of total RNA.

## Saliva fractionation and EV isolation

EVs were isolated by differential ultracentrifugation (Figure 1A). In brief, CFS was centrifuged at 10,000 × *g* for 1 hr at 4°C to pellet MVs. The MV-free supernatant was then transferred to a new tube and ultracentrifuged at 100,000 × *g* for 1 hr at 4°C to pellet EXOs. In this way, 1 mL of EV-depleted saliva (EV-D) was collected along with palleted MVs and EXOs for further RNA isolation. The size and concentration of four saliva fractions were measured by nanoparticles tracking analysis (NTA) with Nanosight NS300 (Supplementary Figure S2).

## RNA extraction

RNA from the four saliva fractions described above (including CFS, MV, EXO, and EV-D) was extracted using Trizol LS (Invitrogen), miRVana (Ambion), MagMAX (Applied Biosystems), Nucleospin (Takara), and miRNeasy Micro (Qiagen), respectively, following the manufacturers’ instructions. The modified miRNeasy method was performed according to the manufacturer’s protocol with specific modifications including glycogen co-precipitant and a second round of chloroform extraction. Briefly, 700 µL of the QIAzol Lysis reagent was added to 140 µL of sample and the mixture was vortexed and incubated at RT for 5 min. Then, 140 µL of chloroform was added to each sample and vortexed for 15 s. The mixture was incubated at RT for 3 min, followed by centrifugation for 15 min at 12,000 × *g* at 4°C. The upper aqueous phase was transferred into a new tube and another 140 µL of chloroform was added, followed by incubation and centrifugation as mentioned above. Then, 1.25× volume of 100% ethanol and 1 µg of glycogen were added to the upper aqueous phase in the second repetition. The mixture was passed through MinElute spin column by centrifugation at 8,000 × *g* for 15 s. The column was then washed with 700 µL Buffer RWT, 500 µL Buffer RPE, and 500 µL 80% ethanol sequentially by centrifugation at 8,000 × *g* for 15 s and finally eluted with 10 μL of RNase-free water at 12,000 × *g* for 1 min.

## Assessment of RNA quantity and quality

Total RNA was quantified using NanoDrop spectrophotometer (Isogen Life Science, Temse, Belgium). RNA purity was assessed by checking the absorbance at 260 nm, 280 nm and 230 nm. To access the RNA integrity, the RNA Integrity Number (RIN) was determined using a 2200 Tapestation Instrument with RNA ScreenTapes (Agilent Technologies, Santa Clara, CA) (Supplementary Figure S3).

## miRNA expression profiling by RT-qPCR

To compare the efficiency of RNA isolation by different methods, RT-qPCR was used to quantify the yield of ten representative miRNAs, including miR-30d, miR-191-5p, miR-27a, miR-26a-5p, miR-21-5p, miR-24-3p, let-7a-5p, miR-92-3p, miR-148a, and miR-320a. The primers used are listed in Supplementary Table S3. Detailed procedures were carried out as described in our previous study [41]. In brief, extracted RNA was reverse transcribed using the High Capacity cDNA Reverse Transcription kit (Applied Biosystems) in a 10 μL reaction. Each reaction was comprised of 1 μL of 10× RT buffer, 0.4 μL of 25× dNTP (100 mM), 0.4 μL of miRNA-specific RT primer mixture (250 nM), 0.5 μL of RNase inhibitor, and 0.5 μL of Multiscribe Reverse Transcriptase. Before the start of RT reaction, reference control sample (i.e. total EVs with no RNA extraction) was incubated for 5 min at 75°C. Then, the RT reaction was carried out using the following condition: 25°C for 20 min, 37°C for 60 min, and 85°C for 5 min. Real-time PCR was performed in a 10 μL reaction including 5 μL of Power SYBR Green PCR master mix (Applied Biosystems) and 250 nm miRNA-specific primers. The reactions were carried out under the following conditions: 95°C for 10 min, followed by 3 cycles of 95°C for 15 s, 45°C for 1 min, and 60°C for 30 s, then 35 cycles of 95°C for 10 s and 60°C for 30 s for data collection. For each miRNA, the mean Ct value was calculated and then compared to that of the control group. The relative changes of the ten miRNAs were averaged to assess the average differences with the control group. For RT-qPCR data analysis, we used the delta-delta Ct method to evaluate expression differences between different RNA isolation methods.

## Small RNA-seq library preparation

Small RNA-seq library preparation was performed using the NEBNext Small RNA-seq Library Preparation Kit with some modifications as previously described [31]. Briefly, 1:10 diluted 3’ SR Adaptor were ligated to the 3’ ends of the RNA, and then hybridized with 1:10 diluted SR reverse transcription primer. Subsequently, 1:5 diluted 5’ SR Adaptor was ligated to the RNA, followed by reverse transcription using ProtoScript II. The reverse transcription products were amplified by 18 cycles of PCR. Then, the PCR products were purified using AMPure XP beads (Beckman Coulter). Amplified cDNA libraries were quantitated with dsDNA Quantifluor (Promega) and sequenced on the Illumina HiSeq platform (with PE read lengths of 2 × 50 base pairs).

## Data analysis

Raw FASTQ RNA-seq data were preprocessed, including trimming of adapter sequences and filtering of low-quality reads, as detailed previously [31]. Subsequently, trimmed reads were mapped to miRBase reference data using Blat to summarize miRNA read counts [42,43]. For tRNA analysis, tRNA reads were aligned to tRFs using the MINTmap pipeline [44]; for yRNA analysis, yRNA reads were aligned to NCBI RefSeq using Bowtie 2 [45]. Next, unmapped reads were further aligned to Kraken microbiome database using the sMETASeq pipeline [46]. The aligned raw reads were normalized to per million reads (RPMs). For pairwise correlation analysis, the log_2_-transformed reads were used to calculate the correlation coefficients across all saliva fractions. Statistical analyses of differentially expressed miRNAs were performed using paired t-test. A *p*-value ≤0.01 with a log_2_ fold change (FC) ≥1 or ≤-1 was considered statistically significant.

## Supplementary Material

Supplemental Material

## Data Availability

The sequencing data are available at NCBI GEO (https://www.ncbi.nlm.nih.gov/geo) under accession GSE222014.
