# Supplementary material for: Characteristics of Human and Microbiome RNA Profiles in Saliva
Source: RNA Biol. 2023 Jul 3;20(1):398–408. doi: 10.1080/15476286.2023.2229596 (PMC10321209; doi:10.1080/15476286.2023.2229596)
Supplement: Supplemental Material [file KRNB_A_2229596_SM6173.zip › Supplementary figures.pptx]

## Slide 1
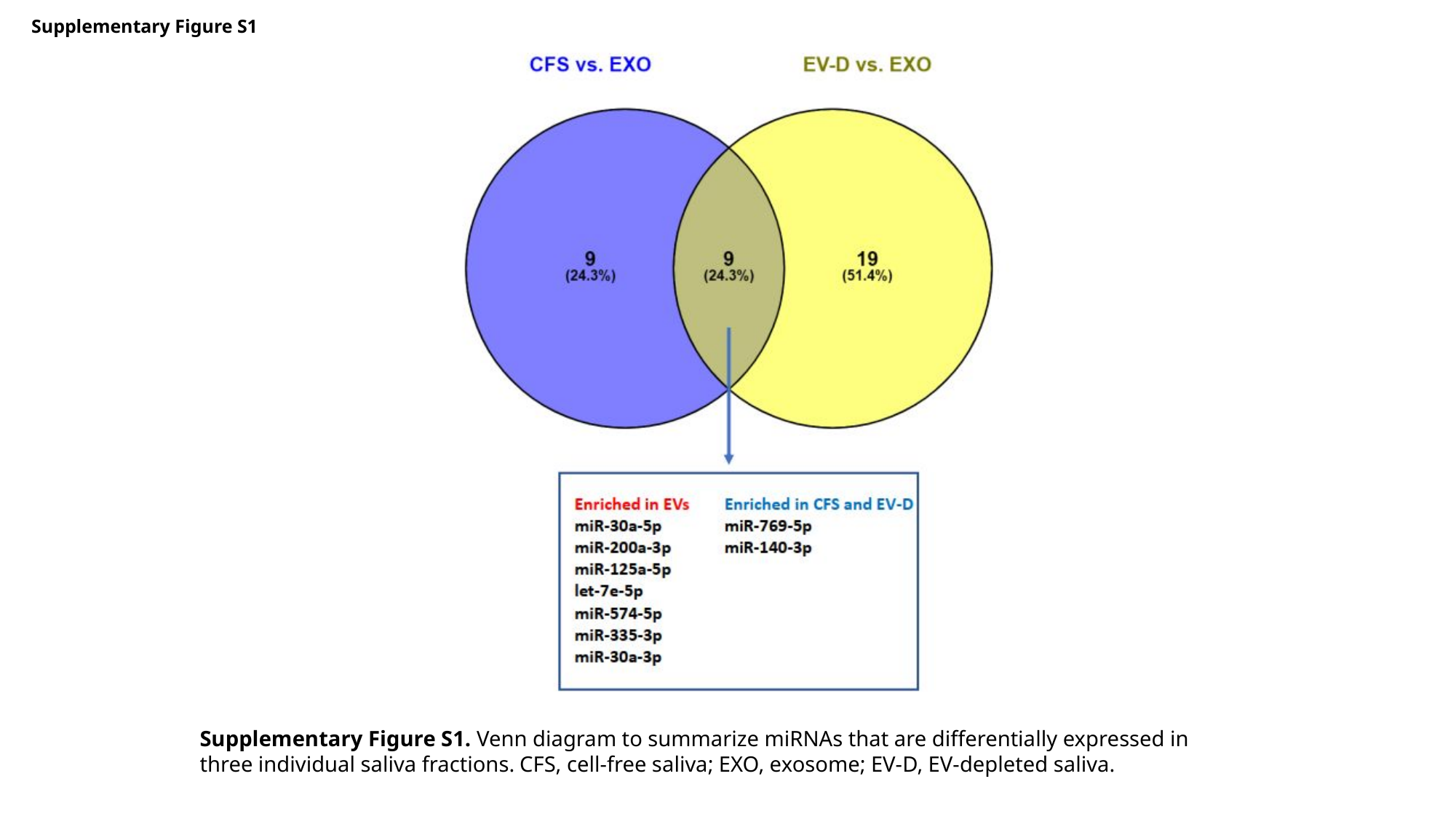

Supplementary Figure S1
Supplementary Figure S1. Venn diagram to summarize miRNAs that are differentially expressed in
three individual saliva fractions. CFS, cell-free saliva; EXO, exosome; EV-D, EV-depleted saliva.

## Slide 2
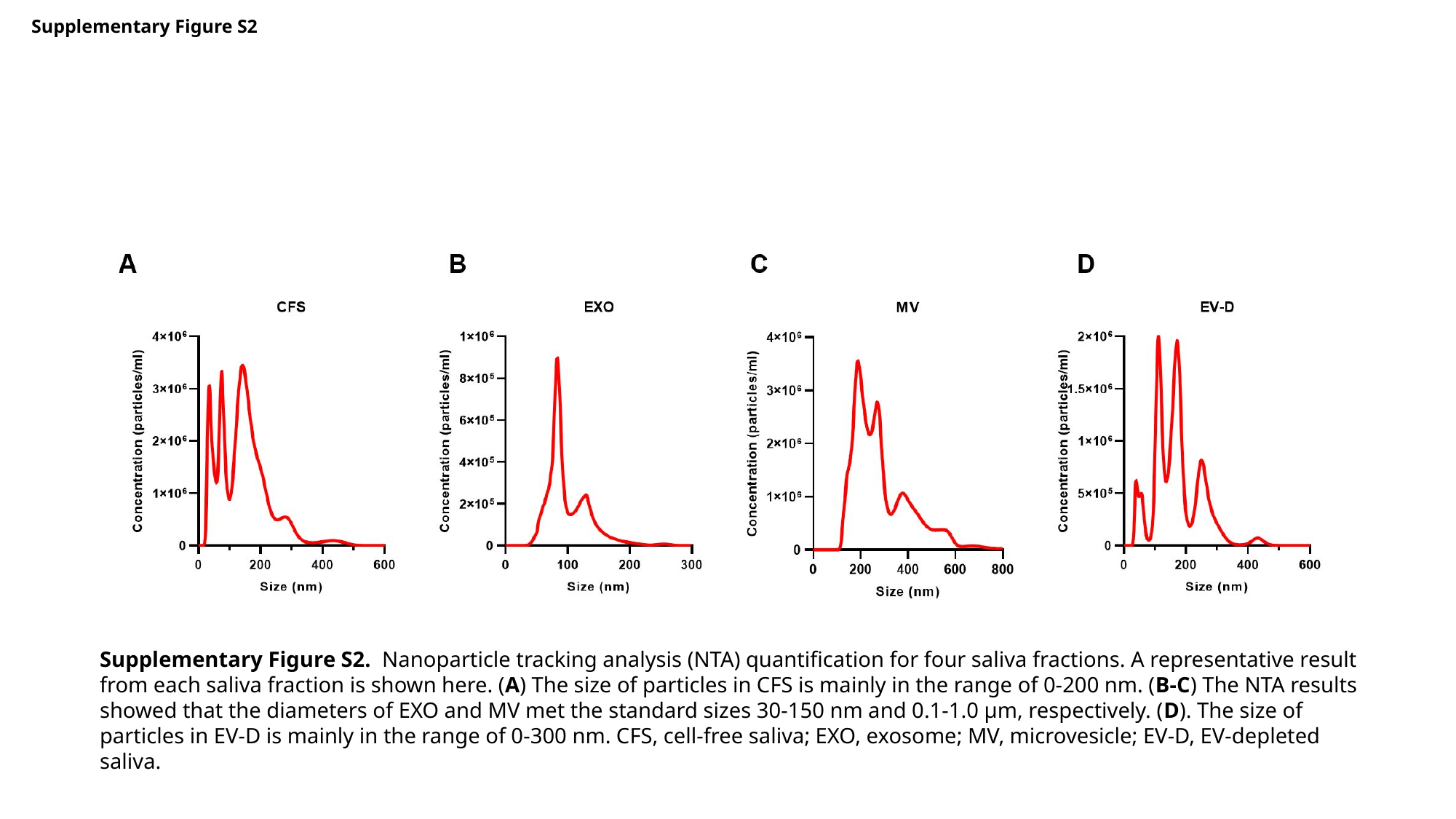

Supplementary Figure S2
Supplementary Figure S2.  Nanoparticle tracking analysis (NTA) quantification for four saliva fractions. A representative result from each saliva fraction is shown here. (A) The size of particles in CFS is mainly in the range of 0-200 nm. (B-C) The NTA results showed that the diameters of EXO and MV met the standard sizes 30-150 nm and 0.1-1.0 µm, respectively. (D). The size of particles in EV-D is mainly in the range of 0-300 nm. CFS, cell-free saliva; EXO, exosome; MV, microvesicle; EV-D, EV-depleted saliva.

## Slide 3
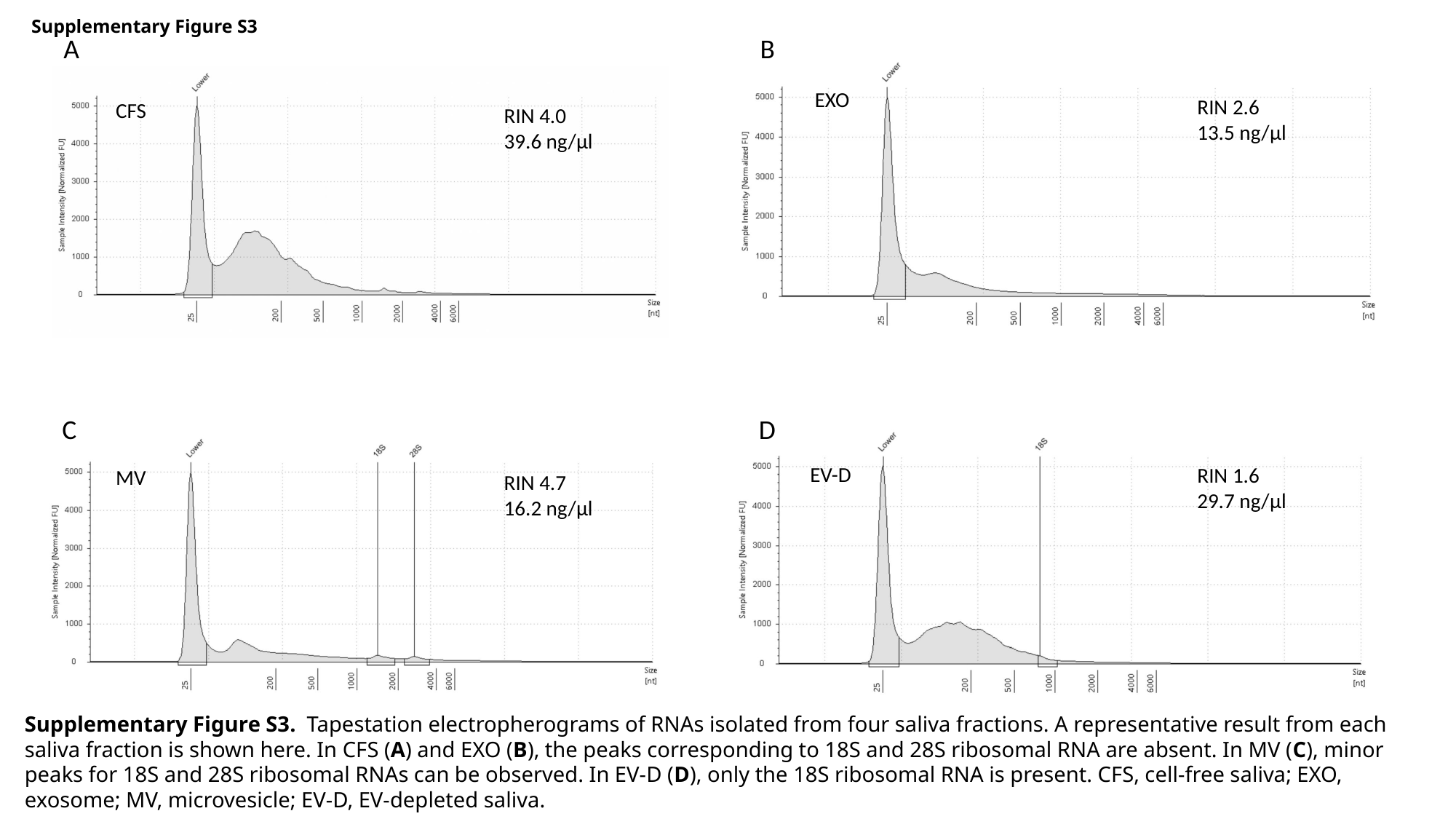

Supplementary Figure S3
A
B
EXO
RIN 2.6
13.5 ng/µl
CFS
RIN 4.0
39.6 ng/µl
C
D
MV
RIN 4.7
16.2 ng/µl
EV-D
RIN 1.6
29.7 ng/µl
Supplementary Figure S3.  Tapestation electropherograms of RNAs isolated from four saliva fractions. A representative result from each saliva fraction is shown here. In CFS (A) and EXO (B), the peaks corresponding to 18S and 28S ribosomal RNA are absent. In MV (C), minor peaks for 18S and 28S ribosomal RNAs can be observed. In EV-D (D), only the 18S ribosomal RNA is present. CFS, cell-free saliva; EXO, exosome; MV, microvesicle; EV-D, EV-depleted saliva.
