# Supplementary material for: Characteristics of Human and Microbiome RNA Profiles in Saliva
Source: RNA Biol. 2023 Jul 3;20(1):398–408. doi: 10.1080/15476286.2023.2229596 (PMC10321209; doi:10.1080/15476286.2023.2229596)
Supplement: Supplemental Material [file KRNB_A_2229596_SM6173.zip › Supplementary table.docx]

**Supplementary Table S1. Differentially expressed miRNAs between EV-D and EXO.**

| **miRNA** | ***P*-value** | **log_2_FC** |
| --- | --- | --- |
| miR-30a-3p | 2.07E-04 | -3.75 |
| let-7e-5p | 3.10E-05 | -3.40 |
| miR-30c-2-3p | 8.70E-04 | -3.40 |
| miR-30a-5p | 8.65E-04 | -2.63 |
| miR-125a-5p | 1.65E-03 | -2.25 |
| miR-574-5p | 2.84E-04 | -2.10 |
| miR-335-3p | 1.17E-03 | -2.03 |
| let-7a-5p | 8.28E-05 | -1.78 |
| let-7f-5p | 6.52E-04 | -1.71 |
| miR-200a-3p | 1.44E-03 | -1.68 |
| let-7c-5p | 2.48E-04 | -1.21 |
| let-7b-5p | 1.34E-04 | -1.18 |
| miR-99b-5p | 4.69E-03 | -1.16 |
| miR-425-3p | 4.24E-03 | 1.09 |
| miR-345-5p | 2.64E-03 | 1.09 |
| miR-10399-3p | 7.52E-03 | 1.11 |
| miR-27a-5p | 1.75E-03 | 1.17 |
| miR-29a-3p | 2.65E-03 | 1.19 |
| miR-23a-3p | 2.21E-04 | 1.20 |
| miR-769-5p | 2.40E-03 | 1.39 |
| miR-652-3p | 5.68E-03 | 1.42 |
| miR-140-3p | 5.27E-03 | 1.47 |
| miR-145-5p | 3.90E-04 | 1.56 |
| miR-15b-5p | 1.77E-03 | 1.57 |
| miR-576-5p | 5.86E-03 | 1.60 |
| miR-425-5p | 3.26E-03 | 1.61 |
| miR-132-3p | 4.39E-03 | 1.65 |
| miR-193b-5p | 3.33E-03 | 1.79 |

**Supplementary Table S2. Differentially expressed miRNAs between CFS and EXO.**

| **miRNA** | ***P*-value** | **log_2_FC** |
| --- | --- | --- |
| miR-141-3p | 7.33E-04 | -3.91 |
| miR-187-3p | 3.98E-03 | -2.88 |
| miR-30a-5p | 3.04E-03 | -2.46 |
| miR-200a-3p | 2.07E-03 | -2.32 |
| miR-885-3p | 7.14E-03 | -2.28 |
| miR-200b-3p | 1.14E-03 | -1.76 |
| miR-125a-5p | 4.24E-03 | -1.75 |
| let-7e-5p | 4.78E-04 | -1.64 |
| miR-574-5p | 2.16E-04 | -1.64 |
| miR-335-3p | 4.71E-04 | -1.56 |
| miR-30a-3p | 2.23E-03 | -1.51 |
| miR-200c-3p | 3.84E-03 | -1.12 |
| miR-200b-5p | 2.77E-03 | -1.06 |
| miR-6842-3p | 4.73E-03 | 1.22 |
| miR-106b-3p | 1.04E-04 | 1.23 |
| miR-769-5p | 1.41E-03 | 1.27 |
| miR-941 | 7.15E-03 | 1.34 |
| miR-140-3p | 1.07E-03 | 1.39 |

**Supplementary Table S3. The sequences of miRNA primers for qRT-PCR.**

| **miRNA** | **primer** | **Sequences (5'-3')** |
| --- | --- | --- |
| miR-30d | RT primer | ctcgtaggatagttgcgaagacttccagtc |
|  | Forward primer | actttcactgtaaacatccccga |
|  | Reverse primer | gctcgtaggatagttgcgaaga |
|  |  |  |
| miR-191-5p | RT primer | cctacgacgcagtaaatcacagctgctt |
|  | Forward primer | tccctacgacgcagtaaatcac |
|  | Reverse primer | gaacacacaacggaatcccaaaa |
|  |  |  |
| miR-27a | RT primer | gaacgaatggaacggctatcgcggaact |
|  | Forward primer | aaattcaggttcacagtggctaag |
|  | Reverse primer | acgaacgaatggaacggctatc |
|  |  |  |
| miR-26a-5p | RT primer | accacacgtcatgtgactgcctatcct |
|  | Forward primer | atcaaccacacgtcatgtgact |
|  | Reverse primer | agcgttgtttcaagtaatccagg |
|  |  |  |
| miR-21-5p | RT primer | cactgtctagcacgacactaatcaacatcag |
|  | Forward primer | ccactgtctagcacgacactaa |
|  | Reverse primer | gccaggcatagcttatcagactg |
|  |  |  |
| miR-24-3p | RT primer | gcacatgactcgtagatacggctgttcctg |
|  | Forward primer | cgcacatgactcgtagatacgg |
|  | Reverse primer | tgcgtggctcagttcagc |
|  |  |  |
| let-7a-5p | RT primer | cgcatatcgcgtcattacagaaactatacaa |
|  | Forward primer | tcgcatatcgcgtcattacaga |
|  | Reverse primer | gcggagttgaggtagtaggttg |
|  |  |  |
| miR-92-3p | RT primer | gccgtcgaggaatcctatcacaggccg |
|  | Forward primer | gagccgtcgaggaatcctatca |
|  | Reverse primer | cgctcttattgcacttgtccc |
|  |  |  |
| miR-148a | RT primer | ccgttcacgatccaaagacacaaagttct |
|  | Forward primer | caaccgttcacgatccaaagac |
|  | Reverse primer | cctcgcttcagtgcactacag |
|  |  |  |
| miR-320a | RT primer | gagcgatagtccgttaggtttcgccctc |
|  | Forward primer | gcgtacaaaagctgggttgaga |
|  | Reverse primer | tggagcgatagtccgttaggtt |
